# Analytical Model of Piezoresistivity for an Inner-Adhesive-Type Carbon Fibre Reinforced Plastic Tunnel Reinforcement

**DOI:** 10.3390/ma15134602

**Published:** 2022-06-30

**Authors:** Hongbin Nie, Shuancheng Gu, Hongmei Mao

**Affiliations:** 1 School of Urban Rail and Engineering, Shaanxi Railway Institute, Weinan 714000, China; 199600112@sxri.net; 2 School of Architecture and Civil Engineering, Xi’an University of Science and Technology, Xi’an 710054, China; gsc5583297@xust.edu.cn

**Keywords:** warning system, surrounding rock pressure, carbon fibre reinforced plastics (CFRP), piezoresistive model, electron tunnelling effect, internode length

## Abstract

Cracks in a tunnel lining often emerge under the coupling action of earth and water pressures in a complex stratum environment, and accidents often occur in the process of repairing cracks. In this study, we used the force-sensitive properties of embedded carbon fibre to conduct early-warning research on lining reinforcement to prevent secondary damage during tunnel lining reinforcement. According to the earth load characteristics, a bond stress–slip model of the embedded carbon fibre under bidirectional earth pressure was established on the basis of the thick-walled cylinder theory and the semi-inverse method in elastic theory. The length change of a single fibre was obtained on the basis of the principle that the volume of a single carbon fibre is constant during the deformation process. The resistance and strain model of the single carbon fibre under the action of an external force was then established following the relationship between the resistance, the length change and the volume change of the single carbon fibre. The resistance of carbon fibre composite materials, according to their production technology and unidirectional force properties, was assumed to be a mixture of the series and parallel resistances of the single carbon fibre, and a piezoresistive model of carbon fibre composite materials was formed by using the multidimensional Taylor series expansion and the idea of the average equivalent. The comparison between the theoretical and monitoring values of the piezoresistive model in a tunnel project in Tibet, China revealed that the resistance of various types of carbon fibres increases with the radius of the lining reinforcement and earth pressure and decreases with an increase in the lining reinforcement thickness. Meanwhile, the angles at different positions of the lining reinforcement also have certain effects on the resistance value of the carbon fibre. The variation curve of the piezoresistive model was exponential in both deeply and shallowly buried tunnels, which verifies the rationality of the model.

## 1. Introduction

In tunnel construction, cracks often occur in the tunnel lining due to geological activities, formation pressure, groundwater and other factors. Two methods are mainly used to prevent and control lining cracks [1,2,3]. The two prevention methods are as follows: one is to set up a telescopic lining according to the size of the formation pressure to facilitate the adaptability of the lining and the formation pressure and the other is to add large-deformation fibre materials to the lining and change the lining stiffness to prevent the generation of cracks [4]. Engineers lack the concept of preventing and controlling cracks and limiting cost; thus, most lining cracks are often controlled using methods such as building a cover arch [5], pouring concrete after planting reinforcement [6] and bonding polymeric plates [7]. The technology of pouring concrete after planting reinforcement is relatively mature; however, this technology destroys the lining structure. Destroying the structure twice before reinforcement is dangerous. The method of using a cover arch is simple and fast, but it occupies considerable space inside the tunnel and affects the normal operation and working space of equipment. The method of bonding the polymeric plate is widely used due to its convenient construction, the preservation of damaged structures and the high strength of the polymer plate. However, in the process of repairing lining cracks, accidents caused by secondary cracking in the lining often occur, which seriously endangers the lives of engineers and technicians [8]. Therefore, scholars at home and abroad have studied this phenomenon and proposed a series of early-warning systems mainly through data acquisition, transmission, extraction and analysis [9,10,11]. Accidents are inevitable, despite the role of lining crack repair [4,12]. Investigation and statistics reveal that data transmission and platform establishment have become advanced after years of development, and research focuses on data acquisition accuracy [13] and the self-sensing properties of cracks over the entire lining [14].

Carbon fibre reinforced polymers (CFRP) are used as a sensing material on account of the electrically conductive carbon fibres and insulating adhesive. The relationship between mechanical deformation and electrical resistance is determined using the iteration model of tandem queues for the internal defect position and size [15]. The coupling capacitance on the adhesive matrix between the fibres is determined through the experimental method of a resistance matrix model [16]. The complete electrode model (CEM) is applied to test the coupling capacitance between the domain and the finite-sized electrodes, but it is not capable of providing electrical conductivity information when the medium possesses highly anisotropic electrical conductivity [17]. The tunnelling effect model, which allows electrons to pass through thin layers of CFRP materials, is developed through the resistance series model [18], and the electrical contact resistance of the interface can be estimated by the generalized Simmons formula [19]. The basic model of carbon fibre and internode resistances in series was proposed on the basis of the microscopic analysis of the test results [20]. The electrical grids in the CFRP were investigated using the MATLAB PDE solver for the conservation law [21]. According to classical physics, the percolation threshold of electrical resistance is below that of percolative-type behaviour. To form conductive concrete, conductive materials including carbon fibre, carbon nanotubes, carbon nanofibres, carbon black, graphite and graphene can be added to the mixture. Through microscopic analysis, it can be seen that the conductive mechanism is mainly due to the change in resistance caused by unidirectional rate sensitivity under the action of external forces [22]. The length and radius of the fibre and the temperature, humidity and other external environmental factors all have a certain influence on the resistance [23]. Carbon fibre test blocks were placed in an electromagnetic coil to measure the electric flux, and related results revealed that the anisotropy of the carbon fibre mixed with concrete results in a defect-self-sensing material prepared using carbon fibre and concrete [24,25,26]. 

The above research leads to the following observations. (1) The structure perception is that the conductive material is added into the structure, and the structure is cracked by an external force, resulting in the change in resistance and then initiating the perception function. (2) To some extent, the piezoresistive model was established through the experimental fitting method, which lacks theoretical support and guidance for engineering applications. The average resistance of the CFRP was tested via series, parallel and mixed models to confirm the inner defect, but the conductive mechanism of the carbon fibre was not studied. (3) The disadvantage of the conductive concrete lies in the anisotropy of the intelligent materials after their addition to the structure, which is manifested in poor resistance perception, low sensitivity and difficulty in determining the resistance threshold.

In this paper, we propose that highly-conductive fibres can be bonded onto the inner surface of the tunnel lining in the form of fabric to reinforce cracks, in order to overcome the disadvantages of anisotropy. Based on the piezoresistive characteristics of the single fibre and the internode characteristics of CFRP, a piezoresistive theoretical model was established to provide warning of the secondary cracking of the lining reinforcement. The relationship between the model and the parameters was analysed, and the feasibility of the model was verified through a comparison with the actual engineering monitoring values.

## 2. Model Establishment

**Figure 1:**
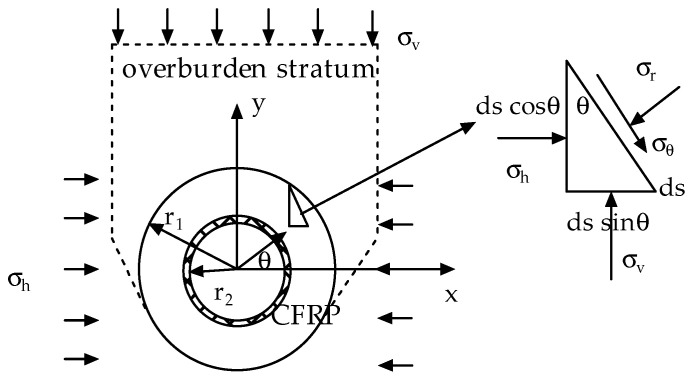
The inner-adhesive-type CFRP for tunnel reinforcement.

A traditional tunnel generally uses a compound lining, which mainly comprises the primary support made of shotcrete and a secondary lining structure made of reinforced concrete. Terzaghi’s consolidation theory of the surrounding rock pressure for a tunnel indicates that vertical gravity pressure will be generated in the overlying rock after the tunnel is excavated, and the horizontal deformation of the soil layer will produce a certain pressure due to the vertical pressure. After the tunnel is supported by the lining, lining cracks occur under the bidirectional action of vertical and horizontal deformation pressures. The embedded carbon fibre fabric is bonded onto the inner-diameter surface of the lining to form lining reinforcement, to prevent the damage caused by secondary cracking. The mechanical model is shown in Figure 1.

## 3. Determination of Pressure Stress of Lining Reinforcement

The triangular force relationship of the lining reinforcement was extracted in accordance with elastic theory, as shown in Figure 1.

The force balance equation in polar coordinates is given in Equation (1).

(1) $$\begin{array}{l} \sum F_{r}=0\;\;\sigma_{r}ds-(\sigma_{h}ds\cos\theta )\cos\theta -(\sigma_{v}ds\sin\theta )\sin\theta =0\\ \sum F_{\theta }=0\;\;\sigma_{\theta }ds+(\sigma_{h}ds\cos\theta )\sin\theta -(\sigma_{v}ds\sin\theta )\cos\theta =0 \end{array}$$

The external load on the lining reinforcement under the bidirectional formation pressure load after sorting is shown in Equation (2),

(2) $$\begin{array}{l} \sigma_{r}{|}_{r=r_{1}}=\frac{1}{2}(\sigma_{h}+\sigma_{v})+\frac{1}{2}(\sigma_{h}-\sigma_{v})\cos2\theta \\ \sigma_{\theta }{|}_{r=r_{1}}=0-\frac{1}{2}\sin(2\theta )(\sigma_{h}-\sigma_{v}) \end{array}$$

where r represents the distance from the centre of the tunnel circle to any point of the lining and the variation range of r is [*r*_2_,*r*_1_]. In addition, *r*_1_ represents the external radius of the lining reinforcement and *r*_2_ represents the internal radius of the lining reinforcement. Assuming that the thickness of the lining reinforcement is t, $\sigma_{r}$ represents the radial stress, $\sigma_{\theta }$ is the tangential stress, $\sigma_{v}$ represents the vertical stress, $\sigma_{h}$ represents the horizontal load and θ represents the angles at different positions of the lining reinforcement.

**Figure 2:**
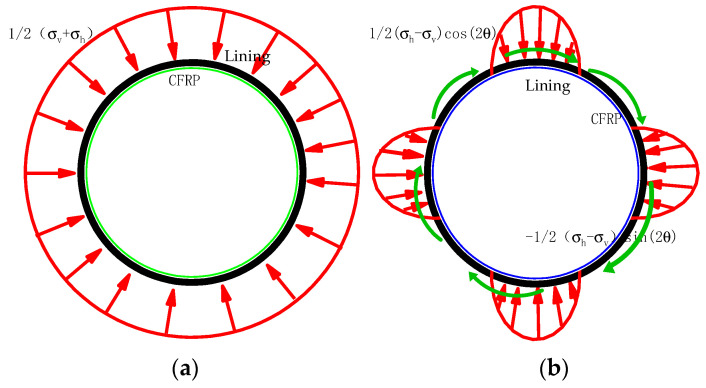
External surface load on lining reinforcement of CFRP. (**a**) Confining pressure action diagram (**b**) Compression–shear combination action diagram with trigonometric functions.

The external load of the lining reinforcement in Equation (2) can be divided into uniformly distributed confining pressure and confining pressure using trigonometric functions, as shown in Figure 2.

According to the thick-walled cylinder theory in elastic theory, the stress of the lining reinforcement when subjected to the uniformly distributed confining pressure shown in Figure 2a is as shown in Equation (3).

(3) $$\left\{ \begin{array}{l} \sigma_{r}=\frac{{r_{1}}^{2}}{{r_{1}}^{2}-{r_{2}}^{2}}\frac{\left(\sigma_{v}+\sigma_{h}\right)}{2}\left(1-{\left(\frac{r_{2}}{r}\right)}^{2}\right)\\ \sigma_{\theta }=\frac{{r_{1}}^{2}}{{r_{1}}^{2}-{r_{2}}^{2}}\frac{\left(\sigma_{v}+\sigma_{h}\right)}{2}\left(1+{\left(\frac{r_{2}}{r}\right)}^{2}\right) \end{array}\right.$$

Figure 2b shows that the trigonometric function for the surrounding rock is solved in accordance with the semi-inverse method of elastic theory [7]. The expression for radial and tangential stresses for external loads contains a trigonometric function; thus, the stress function can be assumed to be as in Equation (4),

(4) ϕ=f(x)cos2θ

where ϕ is the stress function, f(x) is the inclusion function of the stress function and θ is the angle at different positions of the lining reinforcement, which rotates clockwise along the positive direction of the X-axis (Figure 1).

Stress can be expressed by the stress function, as shown in Equation (5).

(5) $$\begin{array}{l} \sigma_{r}=\frac{\partial^{2}\phi }{\partial y^{2}}=\frac{1}{r}\frac{\partial \phi }{r}+\frac{1}{r^{2}}\frac{\partial^{2}\phi }{\partial \theta^{2}}\\ \sigma_{\theta }=\frac{\partial^{2}\phi }{\partial r^{2}} \end{array}$$

This function satisfies the compatibility equation, as shown in Equation (6).

(6) $$\nabla^{2}\nabla^{2}\phi =0$$

The function can be divided into Equation (7) as follows:

(7) $$\frac{d^{4}f}{dr^{4}}+\frac{2}{r}\frac{d^{3}f}{dr^{3}}-\frac{9}{r^{2}}\frac{d^{2}f}{dr^{2}}+\frac{9}{r^{3}}\frac{df}{dr}=0$$

Let $r=e^{t},\;t=\ln(r)$, as shown in Equation (8).

(8) $$\frac{d^{4}f}{dt^{4}}+4\frac{d^{3}f}{dt^{3}}-4\frac{d^{2}f}{dt^{2}}+16\frac{df}{dt}=0$$

The general solutions from the characteristic equation $\frac{df}{dr}$ are 0, −2, 2 and 4.

Then, the expression of the solution is as shown in Equation (9).

(9) $$f(x)=Ar^{2}+Br^{4}+C\frac{1}{r^{2}}+D$$

Equation (9) is substituted into Equation (4) to obtain the stress function, as shown in Equation (10).

(10) $$\phi =(Ar^{2}+Br^{4}+C\frac{1}{r^{2}}+D)\cos2\theta$$

The stress function is substituted into Equation (5) to obtain the stress of the lining reinforcement, as shown in Equation (11).

(11) $$\left\{ \begin{array}{l} \sigma_{r}=-(2A+\frac{6C}{r^{4}}+\frac{4D}{r^{2}})\cos(2\theta )\\ \sigma_{\theta }=(2A+12Br^{2}+\frac{6C}{r^{4}})\cos(2\theta ) \end{array}\right.$$

The boundary condition of the pressure combination trigonometric function when the lining reinforcement material is subjected to bidirectional pressures is shown in Equation (12).

(12) $$\begin{array}{l} r=r_{1}\;\;\;\;\;\left\{ \begin{array}{l} \sigma_{r}=\frac{1}{2}(\sigma_{h}-\sigma_{v})\cos(2\theta )\\ \sigma_{\theta }=-\frac{1}{2}(\sigma_{h}-\sigma_{v})\sin(2\theta ) \end{array}\right.\;\\ r=r_{2}\;\;\;\;\;\left\{ \begin{array}{l} \sigma_{r}=0\\ \sigma_{\theta }=0 \end{array}\right. \end{array}$$

Equation (12) is substituted into Equation (11), and the relationships of the undetermined coefficients of the stress function can be obtained, as shown in Equation (13).

(13) $$\begin{array}{l} 2A+\frac{6C}{{r_{2}}^{4}}+\frac{4D}{{r_{2}}^{2}}=\frac{1}{2}(\sigma_{h}-\sigma_{v})\\ 2A+6B{r_{2}}^{2}-\frac{6C}{{r_{2}}^{4}}-\frac{2D}{{r_{2}}^{2}}=-\frac{1}{2}(\sigma_{h}-\sigma_{v})\\ 2A+\frac{6C}{{r_{1}}^{4}}+\frac{4D}{{r_{1}}^{2}}=0\\ 2A+6B{r_{1}}^{2}-\frac{6C}{{r_{1}}^{4}}-\frac{2D}{{r_{1}}^{2}}=0 \end{array}$$

These equations are then solved simultaneously, and the values of the coefficients to be calculated can be seen in Equation (14).

(14) $$\left\{ \begin{array}{l} A=-\frac{1}{4}(\sigma_{v}+\sigma_{h})\;\;\;B=0\\ C=-\frac{1}{4}{r_{1}}^{4}(\sigma_{h}-\sigma_{v})\;\;\;D=\frac{{r_{1}}^{2}}{2}(\sigma_{h}-\sigma_{v}) \end{array}\right.\;$$

Equation (14) is substituted into Equation (11), and the lining stress for the compression–shear combination under bidirectional substitution can be obtained, as shown in Equation (15).

(15) $$\left\{ \begin{array}{l} \sigma_{r}=\frac{1}{2}(\sigma_{h}-\sigma_{v})\left(1+3{\left(\frac{r_{2}}{r}\right)}^{4}-4{\left(\frac{r_{2}}{r}\right)}^{2}\right)\cos(2\theta )\\ \sigma_{\theta }=-\frac{1}{2}(\sigma_{h}-\sigma_{v})\left(1+3{\left(\frac{r_{2}}{r}\right)}^{4}\right)\cos(2\theta ) \end{array}\right.$$

Equations (3) and (15) are combined, and the stress of the lining reinforcement can be obtained, as shown in Equation (16),

(16) $$\left\{ \begin{array}{l} \sigma_{r}=\frac{{r_{1}}^{2}}{{r_{1}}^{2}-{r_{2}}^{2}}\frac{\left(\sigma_{v}+\sigma_{h}\right)}{2}\left[1-{\left(\frac{r_{2}}{r}\right)}^{2}\right]-\frac{1}{2}(\sigma_{h}-\sigma_{v})\left(1+3{\left(\frac{r_{2}}{r}\right)}^{4}-4{\left(\frac{r_{2}}{r}\right)}^{2}\right)\cos(2\theta )\\ \sigma_{\theta }=\frac{{r_{1}}^{2}}{{r_{1}}^{2}-{r_{2}}^{2}}\frac{\left(\sigma_{v}+\sigma_{h}\right)}{2}\left[1+{\left(\frac{r_{2}}{r}\right)}^{2}\right]-\frac{1}{2}(\sigma_{h}-\sigma_{v})\left[1+3{\left(\frac{r_{2}}{r}\right)}^{4}\right]\cos(2\theta ) \end{array}\right.$$

where $\frac{{r_{1}}^{2}}{{r_{1}}^{2}-{r_{2}}^{2}}$ is the influence coefficient of the geometrical dimensions of the lining reinforcement, which is related to its thickness and radius.

## 4. Piezoresistive Model of Single Carbon Fibre

Assuming that the volume does not change when a single carbon fibre is deformed when subjected to external forces, the length change and cross-section of the fibre are shown in Equation (17),

(17) $$\frac{\Delta r_{cf}}{r_{cf}}=1+\frac{1}{\sqrt{\frac{\Delta l_{cf}}{l_{cf}}+1}}$$

where $\Delta r_{cf}$ is the radius change of a single CFRP, $r_{cf}$ is the radius of a single CFRP, $\Delta l_{cf}$ is the length change of a single CFRP and $l_{cf}$ is the length of a single CFRP.

The deformation of a single fibre is a small change, $\Delta l_{cf}\approx dl_{cf}$. Similarly, in Equation (17), the section coefficient $\nu_{c}$ is introduced to realise an integrable implicit function to represent the function relationship between the rates of length and radius changes.

With $\nu_{c}$, Equation (17) can be converted into Equation (18) of the integrable function,

(18) $$\frac{dl_{cf}}{l_{cf}}=\nu_{c}\frac{dr_{cf}}{r_{cf}}$$

where $\nu_{c}$ is the parameter related to the length and section.

Equation (19) can be obtained by solving the following differential equation,

(19) $$r=e^{\frac{\ln(l)-C_{i}}{\nu }}$$

where $C_{i}$ is the integral constant.

The rate of volume change under the unidirectional tensile stress of the inner CFRP is shown in Equation (20),

(20) $$\frac{dV_{cf}}{V_{cf}}=(1-2u_{cf})\varepsilon_{cf}$$

where $V_{cf}$ is the volume of a single fibre, $u_{cf}$ is Poisson’s ratio and $\varepsilon_{cf}$ is the strain of a single carbon fibre under unidirectional stress $\varepsilon_{cf}=\frac{\Delta l}{l}$.

Assume that the relationship between the electrical resistivity and the rate of volume change is shown in Equation (21),

(21) $$\frac{d\rho_{cf}}{\rho_{cf}}=\alpha_{cf}\frac{dV_{cf}}{V_{cf}}$$

where $\alpha_{cf}$ is the parameter of the electrical resistivity and the rate of volume change.

Equation (22) is obtained in accordance with the rate of the resistance change.

(22) $$\frac{dR_{cf}}{R_{cf}}=\frac{d\rho_{cf}}{\rho_{cf}}+\frac{dl_{cf}}{l_{cf}}-\frac{2dr_{cf}}{r_{cf}}$$

Equations (18) and (20)–(22) are combined to obtain the general solution of the resistance and strain of a single fibre,

(23) $$\;R_{cf}=e^{\left[1-\left(u_{cf}-\nu_{cf}\right)\right]{\varepsilon_{cf}}^{2}+C_{cf}}$$

where $C_{cf}$ is the integral constant of the carbon fibre, which can be written down to facilitate subsequent calculation.

## 5. Piezoresistive Model of Embedded Carbon Fibre

Compared with the radius of the tunnel, the embedded CFRP is much thinner, with negligible thickness. The stress condition of the carbon fibre is $r=r_{2}$, based on the stress equation (Equation (16)) of the lining reinforcement, and the stress equation can be obtained as shown in Equation (24).

(24) $$\left\{ \begin{array}{l} \sigma_{r}=0\\ \sigma_{\theta }=\frac{{r_{1}}^{2}}{{r_{1}}^{2}-{r_{2}}^{2}}(\sigma_{v}+\sigma_{h})+2(\sigma_{v}-\sigma_{h})\cos(2\theta ) \end{array}\right.$$

According to Equation (24), the CFRP is subjected to a tangential, single-direction tensile stress, and the relationship between the stress and strain of the carbon fibre is expressed by Hooke’s Law, as shown in Equation (25).

(25) $$\sigma_{\theta cf}=E\varepsilon_{\theta cf}$$

**Figure 3:**
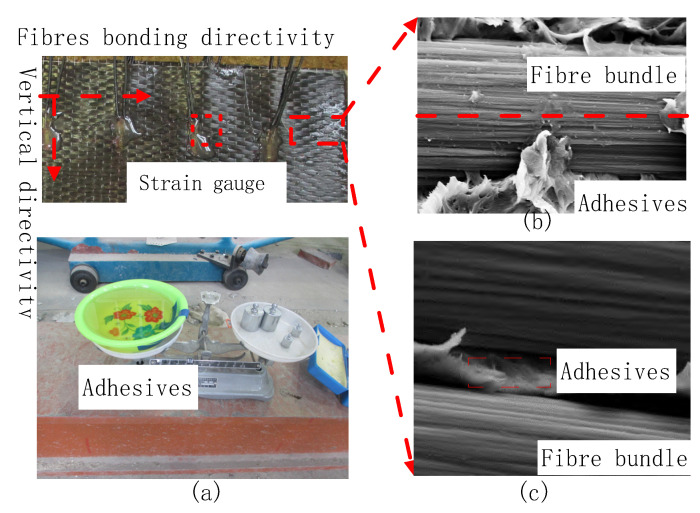
Connection of inner-adhesive-type CFRP: (**a**) operation process of CFRP bonded by adhesives; (**b**) fibre connection along the bond length; (**c**) fibre connection along vertical alignment.

**Figure 4:**
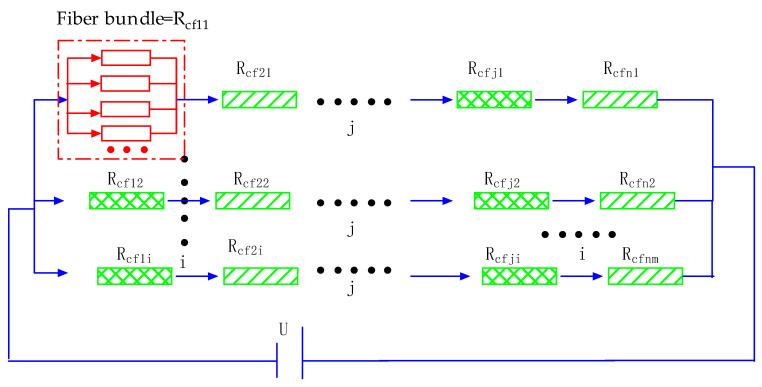
Resistance relationship of the embedded CFRP.

The CFRP is bonded to the inner wall of the tunnel using adhesive, according to the reinforcement specifications. Figure 3 shows the carbon fibre reinforcement diagram for repairing cracks in the Sichuan–Tibet Railway Tunnel. The adhesive is brushed onto the damaged part of the structure with a wooden brush, pasted with CFRP and then repeatedly rolled until the surface of the CFRP sheet is completely wrapped in adhesive, as shown in Figure 3a. The carbon fibre is wrapped in adhesive (an insulating material), according to the microscopic analysis of the carbon fibre composite material performed by Hou Xiangchi. The fibres along the bonding direction of the CFRP are connected in series, as shown in Figure 3b. Meanwhile, the fibres along the vertical and bonding directions are completely wrapped in and separated by adhesive; that is, the fibres are insulated. The vertically oriented fibres in this state are connected in parallel with other fibres, as shown in Figure 3c. The resistance model of the embedded CFRP according to the bonding characteristics of fibres along the length and vertical directions is shown in Figure 4. The equivalent resistance is shown in Equation (26) which has been shown in Appendix A for detailed solution process.

(26) $$R_{cfr}=\frac{1}{\left[\begin{array}{l} \frac{1}{R_{cfz11}+R_{cfz12}+R_{cfz13}+\cdot \cdot \cdot +R_{cfz1j}}+\frac{1}{R_{cfz21}+R_{cfz22}+R_{cfz23}+\cdot \cdot \cdot +R_{cfz2j}}+\cdot \cdot \cdot +\frac{1}{R_{cfzi1}+R_{cfzi2}+R_{cfzi3}+\cdot \cdot \cdot +R_{cfzij}}+\\ \cdot \cdot \cdot +\frac{1}{R_{cfzn1}+R_{cfzn2}+R_{cfzn3}+\cdot \cdot \cdot +R_{cfznm}} \end{array}\right]}$$

The piezoresistive model of the embedded CFRP can be obtained by combining Equations (23) and (26), as shown in Equation (27),

(27) $$R_{cfr}=\frac{1}{\sum_{j=1}^{m}\frac{1}{\sum_{i=1}^{n}e^{\left[1-\left(u_{cf}-\nu_{cf}\right)\right]{\left[\frac{\varepsilon_{ji}}{E}\right]}^{2}}+\sum_{i=1}^{n}C_{cfji}}}$$

where $R_{cfr}$ is the average resistance of the embedded CFRP.

In Equation (27), the Taylor series $e^{\left[1-\left(u_{cf}-\nu_{cf}\right)\right]{\left[\frac{\varepsilon_{ji}}{E}\right]}^{2}}$ is expanded into Equation (28) as follows:

(28) $$e^{\left[1-\left(u_{cf}-\nu_{cf}\right)\right]{\left[\frac{\varepsilon_{ji}}{E}\right]}^{2}}=\sum_{l=0}^{k}\frac{1}{l!}{\left\{\left[1-\left(u_{cf}-\nu_{cf}\right)\right]{\left[\frac{\varepsilon_{ji}}{E}\right]}^{2}\right\}}^{l}$$

$\sum_{i=1}^{n}e^{\left[1-\left(u_{cf}-\nu_{cf}\right)\right]{\left[\frac{\varepsilon_{ji}}{E}\right]}^{2}}$ can then be expanded into Equation (29).

(29) $$\sum_{i=1}^{n}e^{\left[1-\left(u_{cf}-\nu_{cf}\right)\right]{\left[\frac{\varepsilon_{ji}}{E}\right]}^{2}}=n+\sum_{i=1}^{n}{\left[1-\left(u_{cf}-\nu_{cf}\right)\right]}^{l}\sum_{i=1}^{n}\sum_{l=1}^{k}\frac{1}{l!}{\left[\frac{\varepsilon_{ji}}{E}\right]}^{2l}$$

Equation (29) is substituted into Equation (27) to obtain Equation (30).

(30) $$R_{cfr}=\frac{1}{\sum_{j=1}^{m}\frac{1}{\left\{n+\sum_{i=1}^{n}{\left[1-\left(u_{cf}-\nu_{cf}\right)\right]}^{l}\sum_{i=1}^{n}\sum_{l=1}^{k}\frac{1}{l!}{\left[\frac{\varepsilon_{ji}}{E}\right]}^{2l}+\sum_{i=1}^{n}C_{cfji}\right\}}}$$

Equation (30) is expanded by the Taylor series twice according to the form of $\frac{1}{1-x}$, to obtain Equation (31).

(31) $$R_{cfr}=1+\overset{k}{\underset{l=1}{\sum }}{\left\{m+\overset{m}{\underset{j=1}{\sum }}\overset{k}{\underset{l=1}{\sum }}{\left\{n+\overset{n}{\underset{i=1}{\sum }}{\left[1-\left(u_{cf}-v_{cf}\right)\right]}^{l}\overset{n}{\underset{i=1}{\sum }}\overset{k}{\underset{l=1}{\sum }}\frac{1}{l!}{\left[\frac{\varepsilon_{ji}}{E}\right]}^{2l}+\overset{n}{\underset{i=1}{\sum }}C_{cfji}+1\right\}}^{l}+1\right\}}^{l}$$

Fibres are evenly distributed in the embedded carbon fibre sheet. In a certain small range, a minimal difference is observed in the strain between fibres. The strain of a single fibre can be replaced by the average strain at a certain point, as shown in Equation (32).

(32) $$\varepsilon_{jir}\approx \varepsilon_{ji}$$

The average strain at a certain point is calculated in Equation (33),

(33) $$\varepsilon_{jir}=\frac{\Delta l_{\theta }}{l_{\theta }}$$

where $\Delta l_{\theta }$ represents the length variation of carbon fibre along the tunnel section, $l_{\theta }$ represents the length of the carbon fibre along the direction of a section within a certain range and θ represents the angle value of the lining at different positions.

According to the average strain equations of the fibre at a certain point, that is, Equations (33), (24) and (25), the relationship between the strain of the embedded carbon fibres and the external load of the tunnel lining is shown in the equation.

The piezoresistive model of the embedded carbon fibres is obtained by combining Equations (23) and (33), as shown in Equation (34).

(34) $$\varepsilon_{jir}=\frac{\frac{{r_{1}}^{2}}{{r_{1}}^{2}-{r_{2}}^{2}}(\sigma_{v}+\sigma_{h})\;+2(\sigma_{v}-\sigma_{h})\cos(2\theta )}{E}$$

The piezoresistive model of the embedded carbon fibres is obtained by combining Equations (31) and (34), as shown in Equation (35), where *m* represents the assumed number of resistance nodes along the vertical direction of the carbon fibre, *k* represents the expansion coefficient of the Taylor series, *n* represents the assumed number of resistance nodes along the length direction of the carbon fibre, and *i* and *j* represent the process coefficients of the internode resistance. In addition, l represents the process coefficient of the Taylor series and $C_{cfji}$ represents the integral constant.

(35) $$R_{cfr}=1+\sum_{l=1}^{k}{\left\{m+\sum_{j=1}^{m}\sum_{l=1}^{k}{\left\{\begin{array}{l} n+\sum_{i=1}^{n}{\left[1-\left(u_{cf}-\nu_{cf}\right)\right]}^{l}\sum_{i=1}^{n}\sum_{l=1}^{k}\frac{1}{l!}{\left[\frac{\frac{{r_{1}}^{2}}{{r_{1}}^{2}-{r_{2}}^{2}}(\sigma_{v}+\sigma_{h})\;+2(\sigma_{v}-\sigma_{h})\cos(2\theta )}{E}\right]}^{2l}\\ +\sum_{i=1}^{n}C_{cfji}+1 \end{array}\right\}}^{l}+1\right\}}^{l}$$

According to Equation (35), the change in the resistance value of the embedded carbon fibre is related to parameters such as the integral constant, the cross-section coefficient of a single carbon fibre, Poisson’s ratio and the elastic modulus, as well as external factors such as vertical and horizontal formation pressure loads and the angles at different positions of the lining reinforcement.

## 6. Parameter Analysis of the Piezoresistive Model

### 6.1. Correlation Analysis of the Cross-Section Coefficient of the Carbon Fibre

**Figure 5:**
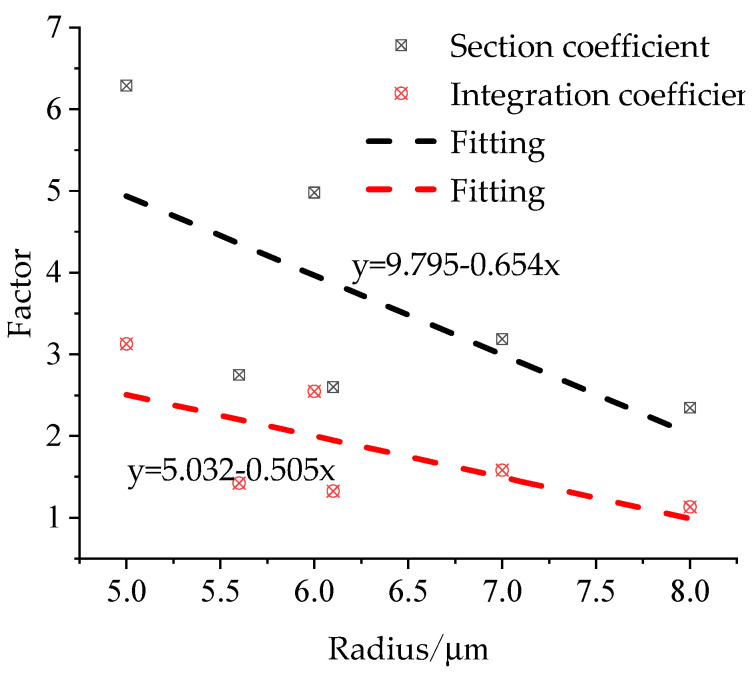
Relationship between fibre coefficient and radius.

Under the action of radial stress, the carbon fibre will be elongated, and its diameter will change correspondingly. This is related to the preparation technology and cross-section coefficient of the carbon fibre. Table 1 shows that the carbon fibres used in the building structure mainly comprise polyacrylonitrile based on dry and wet preparation processes, which can be divided into high-strength and high-modulus carbon fibres. The cross-section coefficient and integral constant of the carbon fibre are calculated by taking 10 mm as an example. The cross-section coefficient is related to the deformation of the carbon fibre and can be expressed by the deformation modulus, which can be calculated using Equation (19). The calculation results are shown in Table 1 and Figure 5. The analysis revealed that the cross-section and integral coefficients of the different fibres gradually decrease with an increase in radius.

**Figure 6:**
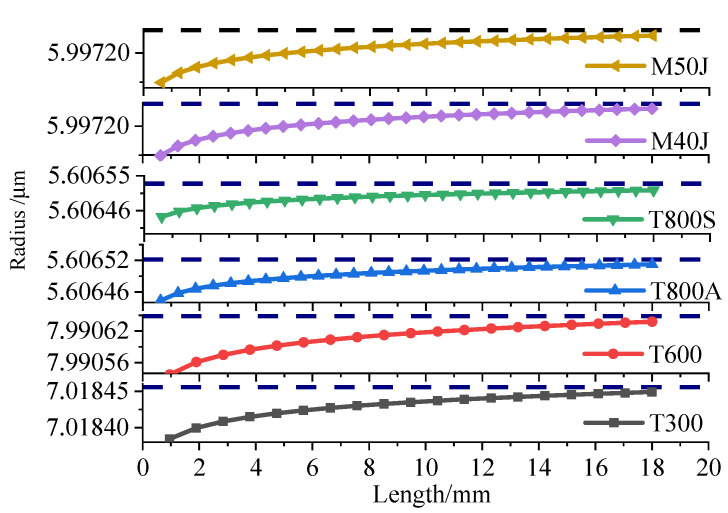
Relationship between the length and width of the carbon fibre under external force.

The relationship between the radius and length of different fibres can be obtained by substituting the cross-section coefficient and integral constant into Equation (20). The fibre with a length of 20 mm is taken as an example, in order to analyse the relationship between length and radius, as shown in Figure 6. Figure 6 indicates that the radius changes exponentially, and the change is fairly small when the fibre length changes from 0 mm to 20 mm. The radius changes slightly, and an extreme value is observed, when the fibre length increases to a certain extent. Some differences are found amongst fibres of different types with different properties, but the index variation trend is consistent.

### 6.2. Analysis of External Load Influence on the Piezoresistive Model

Owing to the tunnel construction, the overlying strata produce vertical pressure, and the soil layer also generates a corresponding horizontal pressure under the action of the vertical pressure. The ratio of vertical to horizontal pressure is called the lateral pressure coefficient, and the radial tensile stress can be expressed as in Equation (36),

(36) $$\sigma_{\theta }=\sigma_{v}\frac{{r_{1}}^{2}}{{r_{1}}^{2}-{r_{2}}^{2}}(1+\lambda )\;+2\sigma_{v}(1-\lambda )\cos(2\theta )$$

where λ is the lateral pressure coefficient and $\frac{{r_{1}}^{2}}{{r_{1}}^{2}-{r_{2}}^{2}}$ is the section coefficient.

Equation (36) shows that the radial stress is related to vertical load, lateral pressure coefficient, geometrical relationship and angles at different positions of the lining reinforcement.

**Figure 7:**
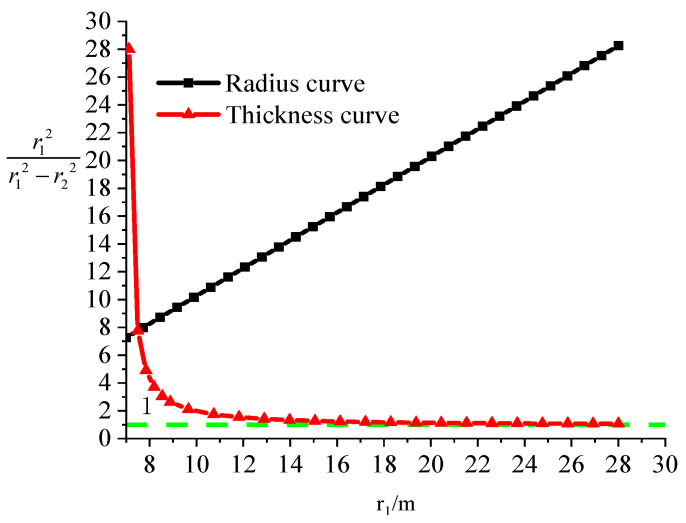
Radius coefficient changes with radius and thickness of the tunnel lining.

The geometric relationship coefficient is related to the radius and thickness of the lining reinforcement, and the analysis is based on the external radius of the lining reinforcement. When the thickness is fixed at 0.5 m, the relationship between the geometric relationship coefficient and the radius can be expressed as the relationship between the geometric relationship coefficient and the external radius, as shown in Figure 7. Figure 7 shows that the geometric parameters increase linearly with the radius. However, the geometric coefficient rapidly decreases with an increase in the thickness of the lining reinforcement, and the geometric coefficient does not decrease and gradually approaches a value of 1 when the thickness of the lining reinforcement is increased to 4 m (where the external radius is 11 m). Therefore, the thickness should be between 0.5 and 1 m in the actual engineering environment, when calculating the preliminary stress of the lining reinforcement (Equation 3), and a certain error exists in calculating the stress of the lining reinforcement using the thick-walled cylinder theory in elastic theory. The error in the stress calculation disappears only when the thickness of the lining reinforcement is larger than 4 m.

**Figure 8:**
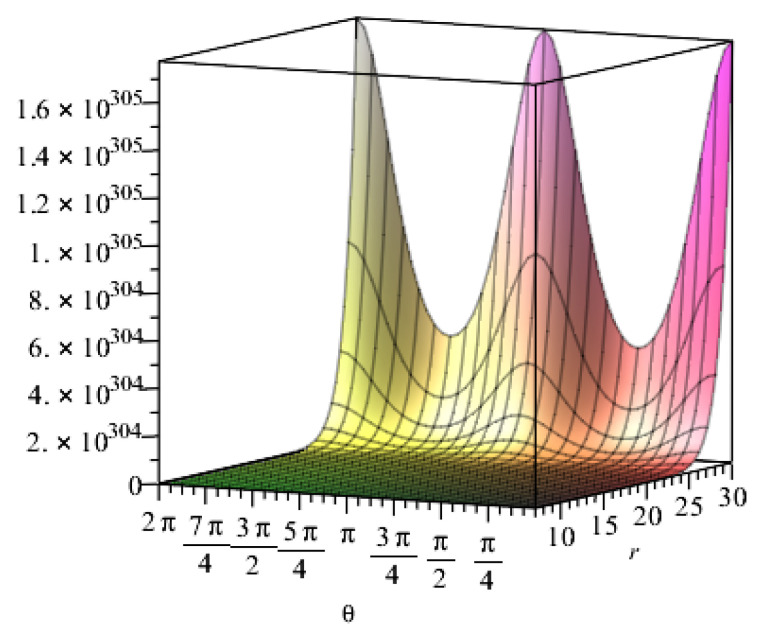
Relationship between the resistance value of the embedded carbon fibre and the radius of the tunnel.

**Figure 9:**
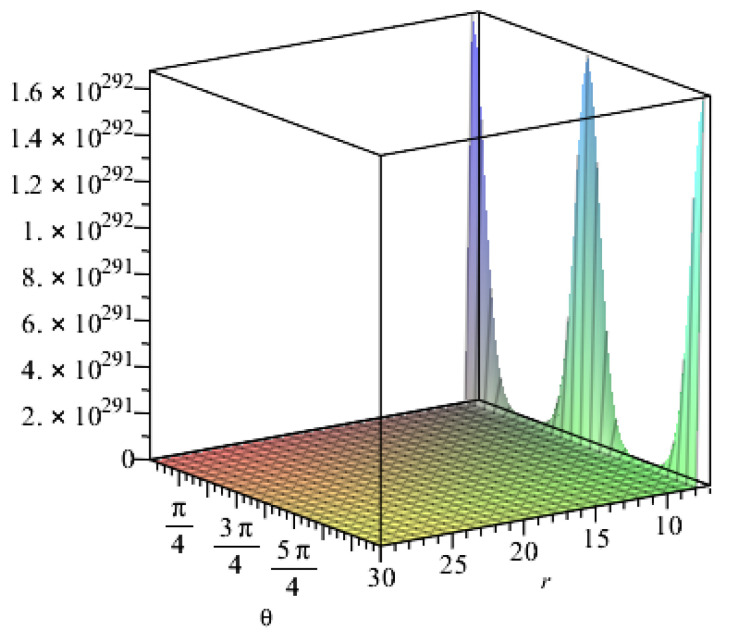
Relationship between the resistance value of the embedded carbon fibre and the thickness of the tunnel.

When other geometric parameters remain unchanged and are positive, the following assumptions are required regarding the influence of geometrical parameters on resistance: the expansion coefficient k in Taylor’s formula is 3; the length of the fibre sheet is 14 m and the width is 0.5 m; the number of fibre internodes along the bonding direction n is set to 200,000; the vertical direction is set to 10,000; the lateral pressure coefficient is set to 0.401; the vertical earth pressure is set to 200 kPa; and Poisson’s ratio is set to 0.307. T300 carbon fibre was then taken as an example, and the section coefficient and integral constant were adopted. Maple software was used to assist the calculation. The resistance value of the embedded carbon fibre changes with the radius and thickness of the tunnel, as shown in Figure 8 and Figure 9, respectively.

Figure 8 shows the relationship between resistance and radius under the influence of the angles at different positions of the lining reinforcement. The resistance value slightly changes and rapidly increases when the radius changes up to 25 m and exceeds 25 m, respectively. In the process of increasing the radius, the resistance value is affected by the angle at different positions of the lining reinforcement, presenting a triangular function distribution. Figure 9 shows the relationship between resistance and thickness. Resistance increases with the thickness when the thickness changes from 0.5 m to 1 m at the beginning. Then, the thickness has minimal influence on resistance despite its continued increase.

**Figure 10:**
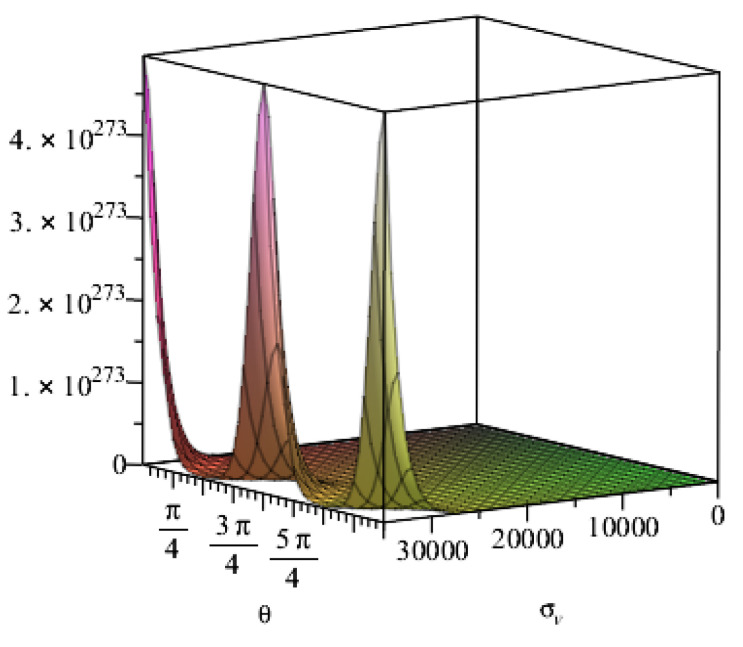
Relationship between vertical stress and resistance.

**Figure 11:**
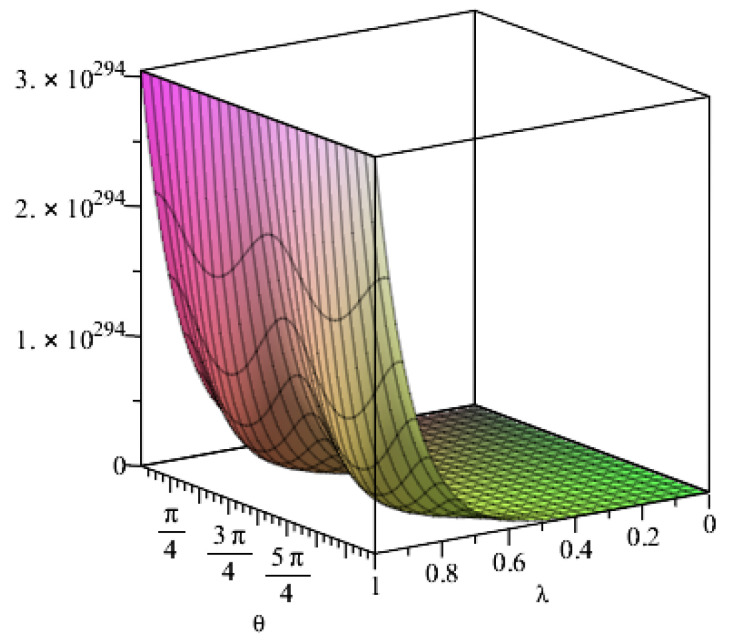
Relationship between lateral pressure coefficient and resistance.

When the vertical pressure is taken as the parameter, the lining thickness is set to 0.5 m, the inner diameter is set to 7 m, the outer diameter is set to 7.5 mm and the lateral pressure coefficient is set to 0.401. The vertical pressure is set to 200 kPa when the lateral pressure coefficient is taken as the parameter. The influence of the vertical and lateral pressure coefficients on the resistance of the carbon fibre can be obtained through calculation, as shown in Figure 10 and Figure 11, respectively. Figure 10 reveals that the resistance value does not substantially change when the vertical pressure is small but varies significantly when the vertical pressure is larger than 200 kPa, and the influence of angles at different positions of the lining reinforcement also changes significantly. Figure 11 shows that when the lateral pressure coefficient is small, the influence on the resistance value is also small, when the lateral pressure coefficient is larger than 0.4 to 0.8 the resistance value increases with the lateral pressure coefficient and when the resistance value continues to increase, the angles at different positions of the tunnel have a substantial influence on the increase in the resistance value. When the lateral pressure coefficient is larger than 0.8, the resistance value rapidly increases and has a linear relationship with the lateral pressure coefficient, which is unaffected by the angles at different positions of the lining reinforcement.

## 7. Application of the Piezoresistive Model in Engineering

**Figure 12:**
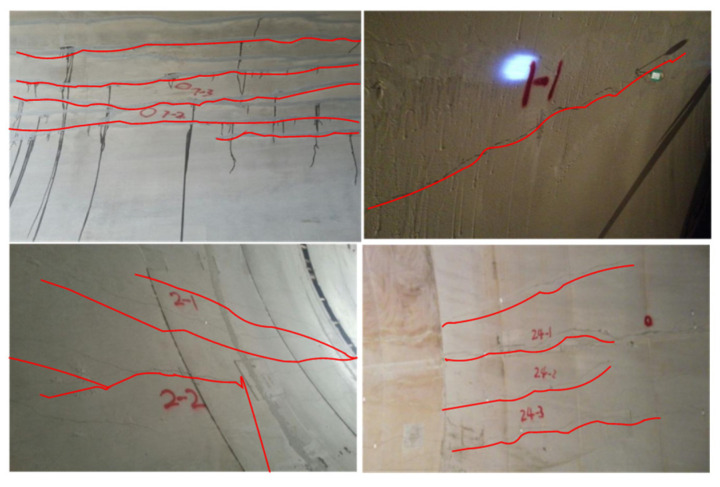
Types of cracks in the tunnel lining.

**Figure 13:**
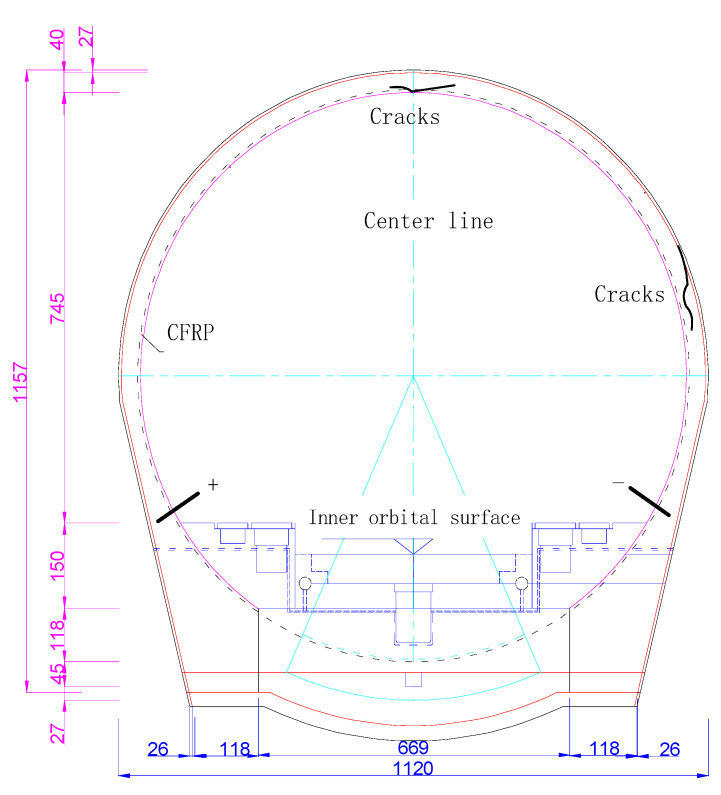
Crack warning method for CFRP reinforced tunnel lining.

During the construction of the Lhasa–Nyingchi Railway tunnel group in China’s Tibet Autonomous Region, part of the lining cracked, and some water seeped through the cracks. Figure 12 shows the location and direction of the cracks. This figure indicates that cracks can be mainly divided into the following types according to their trends: horizontal cracks along the axial direction of the tunnel, circumferential cracks and inclined cracking along the vertical section of the tunnel. The lining has a compound circular lining structure, with the outer diameter set to 5.6 m, the inner diameter set to 5.1 m and the thickness set to 0.5 m. T300 carbon fibre was used to repair the cracks. The mechanical parameters of T300 carbon fibre are shown in Table 2. Construction was performed in accordance with the reinforcement specifications and the Toray carbon fibre manual. Firstly, resin was brushed onto the cracks as the bottom glue to fill the cracks and level the structural plane. A special adhesive was then brushed onto the cracks to level the structural plane and provide bonding for the CFRP. Finally, the CFRP was pasted and repeatedly rolled with a circular stick. This process induces the oozing of adhesive from the surface of the carbon fibre until the adhesive completely immerses the CFRP, thus creating the lining reinforcement. The initial resistance value was measured using the copper rod method when the adhesive had solidified after 24 h. Figure 13 shows the lining structure and the resistance test result for the lining reinforcement.

**Figure 14:**
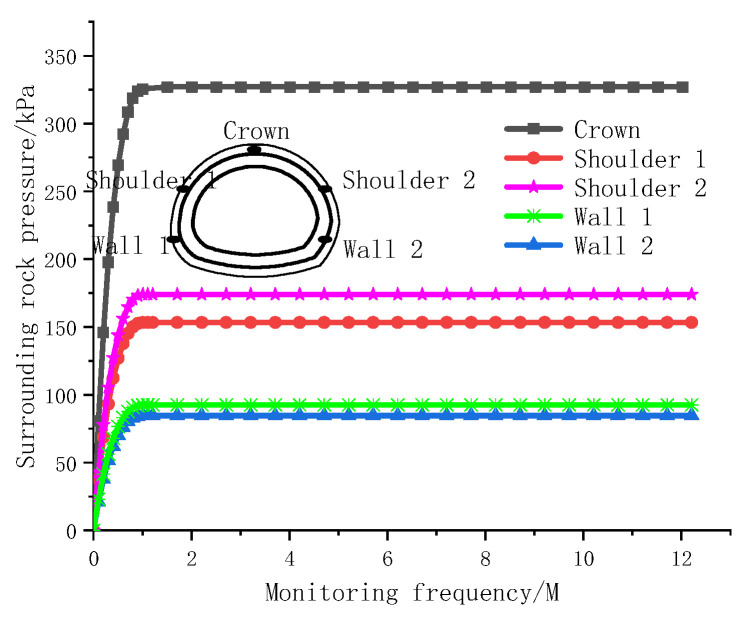
Monitoring values for confining pressures in deeply buried tunnel.

**Figure 15:**
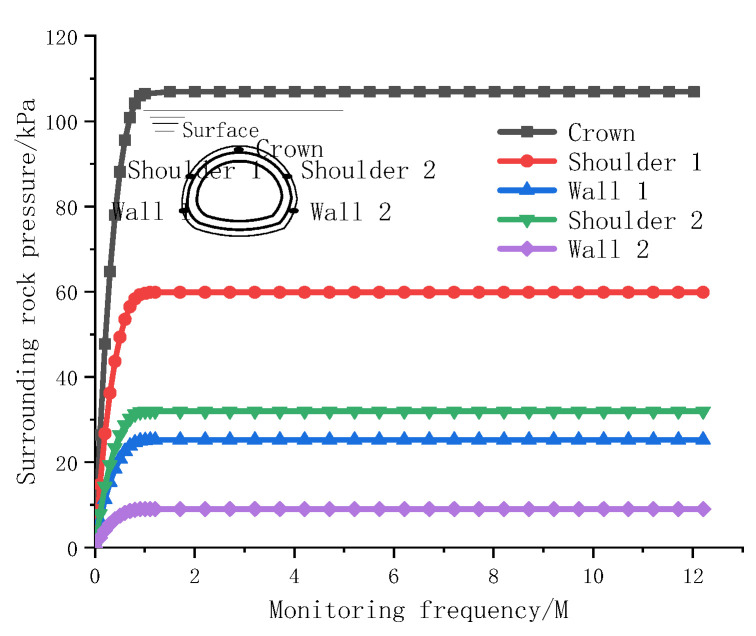
Monitoring values for confining pressures in shallowly buried tunnel.

The pressure of the soil layer around the tunnel is monitored via a pressure box. Pressure boxes are arranged in five measuring positions in the tunnel, including the vault, spandrel and sidewall, to accurately measure and monitor the earth pressure. The vertical and horizontal pressures are represented by the pressures from the tunnel vault and the sidewall, respectively. The tunnel is subjected to different pressures under the action of different strata. The pressure values of the two lining cracks are shown in Figure 14 and Figure 15.

Figure 14 and Figure 15 show that the earth pressure varies with different buried depths, and a certain difference is observed between the horizontal pressure on both sides. Figure 14 shows the earth pressure monitoring value during the construction of deeply buried tunnels. On this basis, the earth pressure increases as the tunnel is deepened. When the earth pressure is redistributed and the tunnel is supported by the lining, the earth pressure is stabilized; the vertical top pressure is as high as 327 kPa, the horizontal pressures on both sides of the soil are similar and small (with an average value of approximately 92 kPa) and the lateral pressure coefficient is 0.281. Figure 15 shows the rock pressure monitoring values for a shallowly buried tunnel. When the vault pressure reaches 110 kPa, the monitoring values tend to converge, when the horizontal pressure of the left wall reaches 30 kPa the monitoring value stabilizes and when the horizontal pressure of right wall 2 reaches 9 kPa, the monitoring value stabilizes and the horizontal pressure difference of the formation reaches 21 kPa. Geological exploration revealed that the horizontal pressure difference was mainly due to a certain inclination angle of the stratum and a certain impact pressure on the lining.

**Figure 16:**
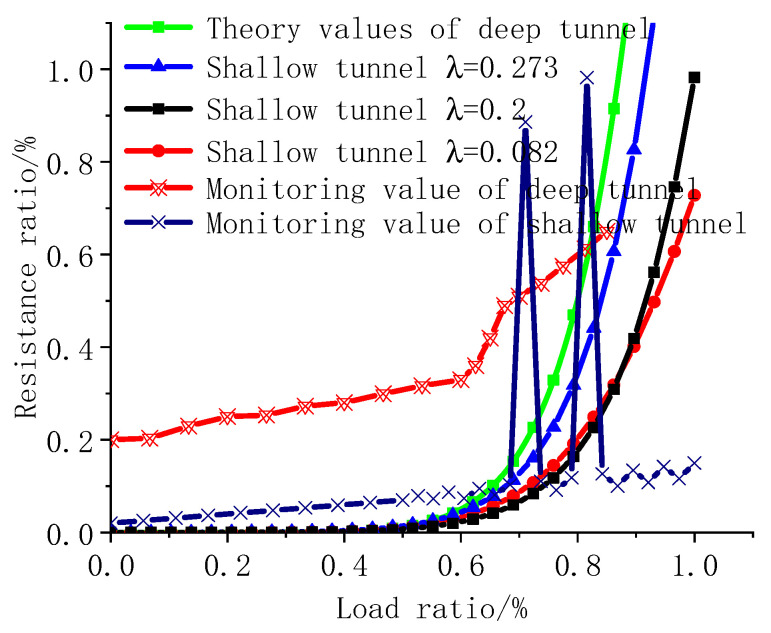
Relationship between theoretically calculated and monitoring values.

The number of internodes n along the fibre length was determined as 2 × 107, according to the length, and the number of internodes n along the vertical fibre bonding direction was set to 10,000. In the deeply buried tunnel, the lateral pressure coefficient was set to 0.281, and the vertical earth pressure was set to 327 kPa. In the shallowly buried tunnels, the vertical pressure was set to 110 kPa, and the lateral pressure coefficient varied from 0.082 to 0.273. The influence of the angles at different positions of the tunnel lining reinforcement was ignored. The tunnel top was taken as an example, and the angle was set to 90°. Under the action of the formation pressure for deeply and shallowly buried tunnels, the relationship between the pressure of the carbon fibre lining reinforcement monitoring value and the theoretically calculated value of the resistance is shown in Figure 16. The analysis of Figure 16 indicates that the resistance and load curves for deeply and shallowly buried tunnels are exponentially distributed. Compared with the shallowly buried tunnel, the piezoresistive curve for a deeply buried tunnel reaches the theoretical extreme value based on resistance theory earlier than for a shallowly buried tunnel. As the lateral pressure coefficient of the shallowly buried tunnel decreases, the piezoresistive theoretical curve becomes increasingly gentle, with a calculated value close to the monitoring value for the shallowly buried tunnels. Regardless of the depth, the theoretical value deviates from the calculated value, and the monitoring value does not reach the theoretical extreme value.

## 8. Conclusions

Intelligent early warning in carbon fibre reinforcement construction was studied on the basis of changes in resistance and pressure in the cracks of the CFRP reinforced lining. The following conclusions can be drawn:

(1) An exponential relationship is observed between the length and radius of a single fibre for different types of fibres.
(2) The resistance value of the lining reinforcement increases with the radius and decreases with an increase in thickness. The influence of angles at different positions of the lining reinforcement can be observed under substantial changes in resistance.
(3) The vertical load of the soil layer affects the lining reinforcement. The resistance value varies with the lateral pressure coefficient when the lateral pressure coefficient fluctuates between 0.4 and 0.8.
(4) The monitoring and theoretical calculation of the resistance and pressure of the carbon fibre reinforced lining in the deeply and shallowly buried sections of the tunnel revealed that the piezoresistive models are exponential in these tunnels, and the resistance monitoring value does not reach the theoretical extreme value.

## Figures and Tables

**Table 1:** Statistics of geometrical parameters of different types of carbon fibre.

Fibre Type	Tensile Strength/MPa	Tensile Modulus/GPa	Ultimate Strain	Diameter/μm	Length/mm	Section Coefficient/10^5^	Integration Coefficient/10^6^	REMARKS
T300	3500	230	0.015	7	10	3.188	1.581	Common
T600	4570	270	0.0016	8	10	2.346	1.133	High strength
T800 A	5638	295	0.019	5.6	10	2.747	1.424	High strength
T800 S	6061	292	0.020	6.1	10	2.601	1.326	High strength
M40J	3880	380	0.012	6	10	4.980	2.548	High modulus
M50J	4120	480	0.014	5	10	6.290	3.128	High modulus

**Table 2:** Mechanical properties of T300 carbon fibre.

Type of Single Fibre	Single Fibre	Tensile Strength/MPa	Tensile Modulus/GPa	Elongation Rate/%	Poisson’s Ratio	Density /(g/cm^3^)
T300	1000	3.530	230	1.5	0.37	1.76

## Data Availability

The data are obtained from mathematical model analysis and engineering monitoring.

## Appendix A

The parallel resistance and the piezoresistive model of the single fibre are expressed in Equations (A1) and (A2), respectively.

(A1) $$\sum_{i=1}^{n}R_{cfzij}=R_{cfz1j}+R_{cfzj2j}+R_{cfz3j}+\cdot \cdot \cdot R_{cfzij}+\cdot \cdot \cdot +R_{cfz\;nj}$$

(A2) $$R_{cfz}=e^{\left[1-\left(u_{cf}-\nu_{cf}\right)\right]{\varepsilon_{cfz}}^{2}}+C_{cfz}$$

The exponential function is expanded by the Taylor series, as shown in Equation (A3).

(A3) $$e^{x}=1+x+\frac{x^{2}}{2!}+\cdots +\frac{x^{l}}{l!}+\frac{x^{l}}{n!}$$

Combining Equations (23) and (33), the exponential function can be expressed as in Equation (A4), after connecting resistances in series.

(A4) $$\sum_{i=1}^{n}e^{\left[1-\left(u_{cf}-\nu_{c}{}_{f}\right)\right]{\left[\frac{\varepsilon_{ji}}{E}\right]}^{2}}=e^{\left[1-\left(u_{cf}-\nu_{c}{}_{f}\right)\right]{\left[\frac{\varepsilon_{j1}}{E}\right]}^{2}}+e^{\left[1-\left(u_{cf}-\nu_{c}{}_{f}\right)\right]{\left[\frac{\varepsilon_{j2}}{E}\right]}^{2}}+\cdot \cdot \cdot e^{\left[1-\left(u_{cf}-\nu_{c}{}_{f}\right)\right]{\left[\frac{\varepsilon_{ji}}{E}\right]}^{2}}+\cdot \cdot \cdot +e^{\left[1-\left(u_{cf}-\nu_{c}{}_{f}\right)\right]{\left[\frac{\varepsilon_{jn}}{E}\right]}^{2}}$$

The exponential function in Equation (A4) is expanded by Equation (A3) (refer to Equation (A5)).

(A5) $$\begin{array}{l} e^{\left[1-\left(u_{cf}-\nu_{cf}\right)\right]{\left[\frac{\varepsilon_{ji}}{E}\right]}^{2}}=1+\left[1-\left(u_{cf}-\nu_{cf}\right)\right]{\left[\frac{\varepsilon_{ji}}{E}\right]}^{2}+\frac{1}{2!}{\left\{\left[1-\left(u_{cf}-\nu_{cf}\right)\right]{\left[\frac{\varepsilon_{ji}}{E}\right]}^{2}\right\}}^{2}+\cdot \cdot \cdot +\\ \frac{1}{l!}{\left\{\left[1-\left(u_{cf}-\nu_{cf}\right)\right]{\left[\frac{\varepsilon_{ji}}{E}\right]}^{2}\right\}}^{l}+\cdot \cdot \cdot +\frac{1}{k!}{\left\{\left[1-\left(u_{cf}-\nu_{cf}\right)\right]{\left[\frac{\varepsilon_{ji}}{E}\right]}^{2}\right\}}^{k}\\ =\sum_{l=0}^{k}\frac{1}{l!}{\left\{\left[1-\left(u_{cf}-\nu_{cf}\right)\right]{\left[\frac{\varepsilon_{ji}}{E}\right]}^{2}\right\}}^{l} \end{array}$$

Equation (A6) can be obtained as shown below after all the indices in Equation (A4) are expressed in accordance with Equation (A5).

(A6) $$\begin{array}{l} \sum_{i=1}^{n}e^{\left[1-\left(u_{cf}-\nu_{cf}\right)\right]{\left[\frac{\varepsilon_{ji}}{E}\right]}^{2}}=1+{\left[1-\left(u_{cf}-\nu_{cf}\right)\right]}^{l}\sum_{l=1}^{k}\frac{1}{l!}{\left[\frac{\varepsilon_{j1}}{E}\right]}^{2l}+1+{\left[1-\left(u_{cf}-\nu_{cf}\right)\right]}^{l}\sum_{l=1}^{k}\frac{1}{l!}{\left[\frac{\varepsilon_{j2}}{E}\right]}^{2l}+\cdot \cdot \cdot +\\ 1+{\left[1-\left(u_{cf}-\nu_{cf}\right)\right]}^{l}\sum_{l=1}^{k}\frac{1}{l!}{\left[\frac{\varepsilon_{ji}}{E}\right]}^{2l}+\cdot \cdot \cdot +1+{\left[1-\left(u_{cf}-\nu_{cf}\right)\right]}^{l}\sum_{l=1}^{k}\frac{1}{l!}{\left[\frac{\varepsilon_{jn}}{E}\right]}^{2l}\\ =n+\sum_{i=1}^{n}{\left[1-\left(u_{cf}-\nu_{cf}\right)\right]}^{l}\sum_{i=1}^{n}\sum_{l=1}^{k}\frac{1}{l!}{\left[\frac{\varepsilon_{ji}}{E}\right]}^{2l} \end{array}$$

The function $\frac{1}{1-x}$ can be expressed by the Taylor series as in Equation (A7).

(A7) $$\frac{1}{1-x}=1+x+x^{2}+\cdot \cdot \cdot +x^{l}+\cdot \cdot \cdot x^{k}$$

Equation (A6) is then expanded by Equation (A7), and the function can be expressed as in Equation (A8).

(A8) $$\begin{array}{l} \frac{1}{n+\sum_{i=1}^{n}{\left[1-\left(u_{cf}-\nu_{cf}\right)\right]}^{l}\sum_{i=1}^{n}\sum_{l=1}^{k}\frac{1}{l!}{\left[\frac{\varepsilon_{ji}}{E}\right]}^{2l}+\sum_{i=1}^{n}C_{cfji}}=\\ \frac{-1}{1-\left\{n+\sum_{i=1}^{n}{\left[1-\left(u_{cf}-\nu_{cf}\right)\right]}^{l}\sum_{i=1}^{n}\sum_{l=1}^{k}\frac{1}{l!}{\left[\frac{\varepsilon_{ji}}{E}\right]}^{2l}+\sum_{i=1}^{n}C_{cfji}+1\right\}}\\ =-1-\left\{\begin{array}{l} n+\sum_{i=1}^{n}{\left[1-\left(u_{cf}-\nu_{cf}\right)\right]}^{l}\sum_{i=1}^{n}\sum_{l=1}^{k}\frac{1}{l!}{\left[\frac{\varepsilon_{ji}}{E}\right]}^{2l}\\ +\sum_{i=1}^{n}C_{cfji}+1 \end{array}\right\}-{\left\{\begin{array}{l} n+\sum_{i=1}^{n}{\left[1-\left(u_{cf}-\nu_{cf}\right)\right]}^{l}\sum_{i=1}^{n}\sum_{l=1}^{k}\frac{1}{l!}{\left[\frac{\varepsilon_{ji}}{E}\right]}^{2l}+\\ \sum_{i=1}^{n}C_{cfji}+1 \end{array}\right\}}^{2}-\\ \cdot \cdot \cdot -{\left\{\begin{array}{l} n+\sum_{i=1}^{n}{\left[1-\left(u_{cf}-\nu_{cf}\right)\right]}^{l}\sum_{i=1}^{n}\sum_{l=1}^{k}\frac{1}{l!}{\left[\frac{\varepsilon_{ji}}{E}\right]}^{2l}\\ +\sum_{i=1}^{n}C_{cfji}+1 \end{array}\right\}}^{l}-{\left\{\begin{array}{l} n+\sum_{i=1}^{n}{\left[1-\left(u_{cf}-\nu_{cf}\right)\right]}^{l}\sum_{i=1}^{n}\sum_{l=1}^{k}\frac{1}{l!}{\left[\frac{\varepsilon_{ji}}{E}\right]}^{2l}\\ +\sum_{i=1}^{n}C_{cfji}+1 \end{array}\right\}}^{k}\\ =-1-\sum_{l=1}^{k}{\left\{n+\sum_{i=1}^{n}{\left[1-\left(u_{cf}-\nu_{cf}\right)\right]}^{l}\sum_{i=1}^{n}\sum_{l=1}^{k}\frac{1}{l!}{\left[\frac{\varepsilon_{ji}}{E}\right]}^{2l}+\sum_{i=1}^{n}C_{cfji}+1\right\}}^{l} \end{array}$$

The sum of Equation (A8) can be expressed as in Equation (A9).

(A9) $$\begin{array}{l} \sum_{j=1}^{m}\left\{-1-\sum_{l=1}^{k}{\left\{n+\sum_{i=1}^{n}{\left[1-\left(u_{cf}-\nu_{cf}\right)\right]}^{l}\sum_{i=1}^{n}\sum_{l=1}^{k}\frac{1}{l!}{\left[\frac{\varepsilon_{ji}}{E}\right]}^{2l}+\sum_{i=1}^{n}C_{cfji}+1\right\}}^{l}\right\}=\\ -m-\sum_{j=1}^{m}\sum_{l=1}^{k}{\left\{n+\sum_{i=1}^{n}{\left[1-\left(u_{cf}-\nu_{cf}\right)\right]}^{l}\sum_{i=1}^{n}\sum_{l=1}^{k}\frac{1}{l!}{\left[\frac{\varepsilon_{ji}}{E}\right]}^{2l}+\sum_{i=1}^{n}C_{cfji}+1\right\}}^{l} \end{array}$$

The equation is continuously expanded in accordance with the $\frac{1}{1-x}$ Taylor series, and Equation (A10) is obtained.

(A10) $$\begin{array}{l} \frac{1}{-m-\sum_{j=1}^{m}\sum_{l=1}^{k}{\left\{n+\sum_{i=1}^{n}{\left[1-\left(u_{cf}-\nu_{cf}\right)\right]}^{l}\sum_{i=1}^{n}\sum_{l=1}^{k}\frac{1}{l!}{\left[\frac{\varepsilon_{ji}}{E}\right]}^{2l}+\sum_{i=1}^{n}C_{cfji}+1\right\}}^{l}}=\\ \frac{1}{1-\left\{m+\sum_{j=1}^{m}\sum_{l=1}^{k}{\left\{n+\sum_{i=1}^{n}{\left[1-\left(u_{cf}-\nu_{cf}\right)\right]}^{l}\sum_{i=1}^{n}\sum_{l=1}^{k}\frac{1}{l!}{\left[\frac{\varepsilon_{ji}}{E}\right]}^{2l}+\sum_{i=1}^{n}C_{cfji}+1\right\}}^{l}+1\right\}}=\\ =1+\sum_{l=1}^{k}{\left\{m+\sum_{j=1}^{m}\sum_{l=1}^{k}{\left\{n+\sum_{i=1}^{n}{\left[1-\left(u_{cf}-\nu_{cf}\right)\right]}^{l}\sum_{i=1}^{n}\sum_{l=1}^{k}\frac{1}{l!}{\left[\frac{\varepsilon_{ji}}{E}\right]}^{2l}+\sum_{i=1}^{n}C_{cfji}+1\right\}}^{l}+1\right\}}^{l} \end{array}$$
